# Results of Some Recent Experiments

**Published:** 1878-10

**Authors:** 


### ARTICLE VI.

### *Results of Some Recent Experiments.*

The more gold there is in a dental alloy, the more oxide of tin will be found at the moment of mixing with mercury.

The black color which stains the hand in mixing an alloy with mercury, is an oxide of tin, almost wholly.

The quickest setting and strongest dental alloys are those made according to their *Chemical equivalents;* and the best amalgam is made by adding mercury according to its chemical equvalent. For instance, the uniting proportion of gold is 197 ; silver 108 ; tin, 58.

Now we can melt together, by weight, 197 parts af gold with 108 parts of silver and 58 parts of tin, or we can use any multiple of these numbers. For instance, we may take 197 parts of gold, which is *one equivalent* of gold ; and 116 parts of tin, which is two equivalents of tin ; and 216 parts of silver, which is two equivalents of silver, and melt them together. In other words, we may take one or more equivalents of either of these metals and melt them with any other whole equivalents of the metals mentioned. Suppose we make a dental alloy of gold 1, silver 3, tin 6, not by weight but by *chemical equivalents,* then we must amalgamate it with mercury 4, or four equivalents of mercury. The formula of the resulting amalgam would be as follows, according to weight:

Gold, 197.
Silver, 324.
Tin, 348.
Mercury, 400.

Now, it will be found in practice that this quantity of mercury is exactly the best quantity to use. This would be to 100 parts of filling, 46 parts, by weight, of mercury. If more mercury is used, all excess would be retained in the amalgam mechanically, and not united chemically with the other metals. Consequently, it is liable to leave the plug by slow evaporation.

When I have melted silver and tin together in the usual proportions of dental alloy, say 4 parts (by weight) of silver to 5 parts of tin, I find the ingot uneven in texture and metalic composition. Some portions contain more silver or more tin than another portion. This I attribute to those portions having no partners with which to unite. Say I take 432 silver, by weight, which is exactly 4 chemical equivalents of silver, and melt with it 540 parts, by weight, of tin. This is, then, the usual formula made of 4 silver, 5 tin. Let us see now if the tin is in exact chemical proportions. As the chemical equivalent of tin is 58, we can find how many *equivalents* of tin there are in the 540 parts by dividing that number by 58. We find there are nine chemical equivalents of tin and 18-58 over. Now, as these molecules of tin have no molecules of silver to unite with, they are merely retained in the ingot mechanically. If we made a formula 4.5 silver, 5.5 tin, then there would be an excess of only 6-58 of tin.

Suppose an alloy containing this excess is amalgamated for a dental plug ; then we have two kinds of amalgam in the same plug, one of silver and tin *chemically united*, and one of the surplus tin alone. The effect of this, *practically*, is to make the plug less tenacious or strong.

One formula of a gold alloy which I published as being good, was composed by weight of gold 1, tin 1, silver 1. Let us see now how this corresponds to the chemical proportions necessary to make a chemical formula.

Gold 197, 1 chem. equiv.

Silver 197, 1, 89–108 chem. equiv.

Tin 197, 3, 23–58 chem. equiv.

In this alloy, then, there were 89 equivalents of silver and 23 of tin that could not unite with gold, but a large portion of the remaining tin united with the whole of the silver, leaving a small surplus of tin to unite only with the mercury in amalgamation. Now here are three kinds of amalgams in the same plug; and we have more mechanical mixture than in the silver and tin alloy, and consequently a less strong plug. Still we have a plug that does not contract in hardening. Therefore, it makes a water-tight plug.

There are some alloys that do not contract when made according to chemical proportions. But all formulas in chemical proportions may not result so. An alloy thus made is much harder and stronger when thus made than one not so. It is very lately that I have found this out.—*Dr. Chase, in Dental Quarterly.*

---

